# FINDING YOURSELF: PERSONALIZED MEDICINE, DATA SCIENCE, AND INTERACTIVE VISUALIZATIONS

**DOI:** 10.1093/geroni/igac059.711

**Published:** 2022-12-20

**Authors:** Michael Griswold, James Henegan, Chad Blackshear, Beverly Gwen Windham, Thomas Mosley

**Affiliations:** The MIND Center at UMMC, Jackson, Mississippi, United States; UMMC MIND Center, Jackson, Mississippi, United States; UMMC MIND Center, Jackson, Mississippi, United States; UMMC-The MIND Center, Jackson, Mississippi, United States; UMMC-The MIND Center, Jackson, Mississippi, United States

## Abstract

Personalized medicine is care that is tailored to an individual patient. In contrast, randomized trials are designed to report evidence of benefits and harms “on average”. The average trial participant though, is often healthier than older patients seen in clinical practice. A variety of methods have been proposed which offer improvements to traditional (but questionable) practices of one-subgroup-at-a-time examinations of treatment effect heterogeneity, but translating evidence from these advancements has received less attention. Data Science initiatives have allowed broader data sharing, data harmonization and data synthesizing approaches, as well as an enormous maturing of interactive visualization techniques. Motivated by the SPRINT study and using advanced approaches of marginalized standardization and the Predictive Approaches to Treatment effect Heterogeneity (PATH) statement, we show how researchers, regulators, clinicians and patients can "find themselves" on the treatment effect continuum and be better informed of potential individualized evidence through interactive visualizations.

